# Society Meetings

**Published:** 1913-06

**Authors:** 


### SOCIETY MEETINGS.

*Brief reports and announcements of meetings of societies of Western New York and of those of general scope are requested from secretaries. Copy should be on hand by the fifteenth of each month. Full transactions will be published at cost of composition.*

A regular meeting of the MEDICAL SOCIETY OF THE COUNTY OF ERIE was held in the Buffalo Library Building on April 21st, 1913, at 8:40 p. m., President Whitwell presiding.

Minutes of previous meeting held February 17th, 1913, were read and approved, as were also the minutes of the Council meetings of March 3d and April 7th, 1913.

Dr. Bonnar, Chairman of the Board of Censors, made his report.

On motion of Dr. McKee the foregoing report was adopted.

No reports were received from chairmen of standing committees.

Dr. William H. Thornton, Chairman of the Special Committee on Collection of Accounts, made a partial report.

The committee had postal cards printed and sent to the members of the society. About 30 per cent. of the members replied.

Committee, therefore, reported progress and was given further time.

Dr. Wall said that, at the last meeting of the A. M. A., a proposal was made for changing the by-laws covering three classes of members. He moved that it is the sense of this society that the proposed new amendments to the by-laws making three classes of members in the A. M. A. be approved.

Dr. Bennett asked if this motion carried with it instructions to delegates to the State Society to that effect. Dr. Wall replied that it would. Dr. Bennett then stated that he would be opposed to the motion for the reason that the society knew too little about this matter to instruct its delegates as to how to vote.

Dr. McKee moved as an amendment that the entire matter be referred to the delegates to the State Society.

Dr. Wall accepted this amendment, which was then carried.

Dr. Crego moved that a committee of three be appointed to investigate the question of fees for examination in lunacy and report at the next meeting of the society. Carried.

Dr. McKee said that the next meeting of the State Society would be held in Rochester next week; that this was the first time in the history of the State Society that it has met outside of Albany. He, therefore, moved that the Secretary be instructed to get out postal cards to members reminding them of this meeting and urging their attendance.

This completed, the regular business and the scientific program was taken up, as follows:

A paper on "Air, Water and Salts," by Dr. Henry R. Hopkins.

A paper on "Sane or Insane," by Sydney A. Dunham.

Both papers were discussed by the members present, after which the meeting adjourned.

FRANKLIN C. GRAM, Secretary.

(Received too late for last issue.)

---

**Report of Progress of the Buffalo Society of Sanitary and Moral Prophylaxis.**

One of the most encouraging and enlightening efforts which have been made of late years for improved sanitation is that which has for its object the restriction of the ravages of venereal diseases.

Hardly more than half a dozen years ago physicians began to organize for this purpose. Later they were joined by influential laymen, and now organizations called Societies of Sanitary and Moral Prophylaxis, or by some similar name, are scattered throughout the United States. The Buffalo branch of this society held its annual meeting recently, and the report of progress presented by the President, Dr. Lucien Howe, we publish in full.

It is due to the members of this Society, and it may be of some interest to affiliated workers, to know what the Society has accomplished during the past year.

As one of our principal objects is to awaken in the public mind an appreciation of the importance of sex hygiene, and to make known the terrible results of venereal diseases, it has therefore seemed better to accomplish this end by teaching. For this purpose the following lectures have been delivered:

At the University of Buffalo—February 2d, Dr. F. W. Barrows, "Sanitary and Moral Prophylaxis." To men. February 9th, Dr. Rosalie S. Morton of New York, "Sanitary and Moral Prophylaxis." To women.

At the Twentieth Century Club—February 6th, Dr. Lucien Howe, "Race Improvement and Better Breeding." February 20th, Dr. Herman G. Matzinger and Miss Frances Ney, "Who Are the Mentally Defective, and What to Do About It?" March 7th, Dr. Helene Kuhlman, "Mental Hygiene."

Dr. Rosalie S. Morton came partly at the request of the officers of this Society, and of the University Committee on Public Lectures, but especially as the representative of the State Board of Health.

In addition to these lectures more informal addresses on sex hygiene and related topics have been given by physicians and other members of the Society at schools, and before small organizations. Competent speakers and teachers can always be furnished free of charge to groups interested in the subject. Mothers are ready to give other mothers facts which daughters should know, and fathers tell other fathers how and why their sons should not start out blindfolded to meet temptation, disease, suffering and sometimes early death.

The reasons for the existence of such a society as this have been stated too often to be repeated here. It is only necessary to recall the fact, for example, that over fifty per cent. of the blindness is due to venereal disease. The surest and best way to prevent blindness is to remove this cause. All others are secondary.

It may be mentioned also that New York pays several hundred thousand dollars each year for insane patients in its hospitals suffering from one disease only—syphilitic paresis. These and other examples of which we are gradually learning could be multiplied indefinitely; the public is awakening to the importance of the subject.

It is only about eight years ago that the first Society of this kind was formed in America. Since then State societies, having the same objects and usually the same name, have been organized in Pennsylvania, Maryland, Missouri, Indiana, Michigan, Colorado and other states, and numerous local branches are being established.

People as a whole are beginning to realize that our schools teach physiology but cannot teach sex hygiene; our pulpits teach morals, but many essential points cannot be dealt with there. Meanwhile married women are constantly exposed, largely because of the ignorance of their husbands, and our sons are in constant danger physically and morally. A Society of Sanitary and Moral Prophylaxis is therefore in a certain way a clearing house for valuable energies, too often pent up, or not well directed. In its educational department it aims to teach parents how to teach their children, also to prevent spread of disease, and to make practical application of the lessons from the pulpit.

The dues of a dollar a year are trifling, and the work required of each family is usually such as coincides with the natural duties of a parent, or is in accord with the member's profession or other occupation. If men and women find it worth while to sustain schools, hospitals and churches, is it not also worth while to add numbers and strength to a society like this in Buffalo, as has been done in many other cities, and thus push forward the movement for cleaner and better living?

The American Proctologic Society will hold its fifteenth annual meeting at the Hotel Radisson, Minneapolis, June 16 and 17, under the presidency of Dr. L. J. Hirschman of Detroit. The Secretary is Dr. L. H. Adler of Philadelphia. There are 34 active fellows, 6 honorary, 6 associates and 7 candidates for membership. None is from our special territory. An interesting program of twenty papers is announced.

---

The Medico-legal Society held a jubilee meeting at the Waldorf-Astoria in New York May 21, to commemorate the thirtieth anniversary of the founding of the *Medico-Legal Journal.* The President is Dr. T. D. Crothers of New Haven and the Secretary and Editor of the *Journal* is Mr. Clark Bell, who was President of the Society forty years ago.

---

The Monday Club of Rochester held a dinner April 29 at which Dr. Howard Kelly of Baltimore gave an address on the control of the social evil in his city. The President, Dr. John R. Williams, acted as toastmaster.

---

The annual meeting and dinner of the American Medical Editors' Association will be held at the Hotel Radisson, Minneapolis, June 16.

---

The sixteenth annual meeting of the American Gastro-enterologic Association was held in Washington, May 5 and 6. The members were entertained at luncheon by Dr. William Gerry Morgan of Washington.

---

The Elmira Academy of Medicine met May 7. Papers were presented by Dr. M. L. Bennett of Watkins on "Abscess of the Liver; by Dr. A. W. Booth of Elmira on the "Significance of Haemorrhage from the Mucous Membranes"; by Dr. R. B. Howland of Elmira on "Erysipelas with Rare Complications."

---

The Women's Medical Society of New York State met in Rochester just before the Medical Society of the State of New York. Two hundred women were in attendance. Dr. Marion Craig Potter of Rochester was elected President, Dr. Helene J. C. Kuhlman of Buffalo retiring.

---

A regular meeting of the Rochester Pathological Society was held April 3d, 1913. Reader, Dr. Thos. G. Tousey. Subject, "Secondary Anaemias." Great stress was laid on poor sanitary conditions as being one of the most important causes

(bad air, light, etc.) It was Dr. Tousey's opinion that these causes acted most probably through products of auto-intoxication inhibiting blood formation in the bone marrow.

At the regular meeting April 17th, 1913, the program was: Reader, Dr. H. L. Prince. Subject, "The Abbott Treatment of Lateral Curvature of the Spine." Several lantern slides were shown, showing the marked benefits and improvement over the old treatment.

---

The Spring meeting of the Hospital Medical Society of Rochester was held at the Oak Hill Country Club on Thursday evening, May 22, 1913, at 8:45 o'clock. Speaker, Professor H. L. Fairchild of the University of Rochester, subject, "Maps Showing the Later Glacial History of New York State."

---

Medical Society of the County of Monroe. Regular May meeting held on May 20, 1913, at the rooms of the Rochester Academy of Medicine. The principal business consisted in the election by ballot of a list of fourteen experts in insanity for one year, which had been nominated by the Board of Censors. The list of experts is sent to every court and lawyer in Rochester and Monroe County with a brief note stating that the Society vouches for them. In this simple way, our profession may be able to force a reform in medical expert testimony. This method was proposed by Dr. S. W. Little and put in operation during his term as president of our County Society. No one is obliged to use these experts, but if they are not employed then the medical profession cannot be blamed because of medico-legal scandals. The program consisted of a paper by Dr. M. May Allen on "Methods of Teaching Sex Hygiene."

C. W. HENNINGTON, Secretary.

---

The Western N. Y. Homeopathic Medical Society, at its meeting in Rochester, April 12, elected the following officers: President, George R. Critchlow of Buffalo; vice-Presidents, Frank Warner of Canandaigua and W. H. Vosburg of Dunkirk; Secretary-Treasurer, R. Montford Schley of Buffalo.

---

The Aesculapian Club of Buffalo recently celebrated its fifteenth anniversary by a dinner at the Buffalo Club, the President, Francis M. O'Gorman, acting as toastmaster.

---

The American Academy of Medicine will hold its 38th annual meeting at the Leamington Hotel, Minneapolis, June 13-15. A large number of papers dealing with education, child hygiene, sociology, etc., is promised. The profession is invited.

The Rochester Academy of Medicine, Section of General Medicine, met May 14. Dr. Allen A. Jones of Buffalo gave a lantern slide demonstration of Gastro-Intestinal cases. Dr. Jones was entertained at dinner at the Rochester Club.

---

The Buffalo Academy of Medicine. Announcements of officers elected for the Academy and the Sections will be made in our next issue, and such general announcement as is available will be made during the summer of the papers scheduled for the coming year.

---

The Medical and Surgical League had its annual meeting and dinner at the Statler Hotel May 8th. Mr. Ernest McIntyre delivered an address on "Medical Expert Testimony." The following officers were elected: President, Dr. Julius Richter; vice-President, Dr. George Seitz; Secretary, Dr. Frank Kleckner; Treasurer, Dr. Edmund Reimann, and Governors, Drs. A. W. Hengerer, George Stesel, Robert Taylor, W. H. Brauns and F. M. O'Gorman.

